# Editorial: Methodological issues in psychology and social sciences research

**DOI:** 10.3389/fpsyg.2022.1111056

**Published:** 2023-01-05

**Authors:** Begoña Espejo, Marta Martín-Carbonell, Irene Checa

**Affiliations:** ^1^Department of Behavioral Sciences Methodology, Faculty of Psychology, University of Valencia, Valencia, Spain; ^2^Faculty of Psychology, Cooperative University of Colombia, Bogotá, Colombia

**Keywords:** psychometrics, simulation studies, item response theory, meta-analysis, systematic review, testlet response theory, Bayesian statistics, statistical significance

In recent decades, the classical methods of studying the psychometric properties of tests have advanced to evolve into new innovative statistical methods, which allows obtaining a new view of the data. In this sense, progress has been made in the use of item response theory and in the use of structural equation models. Likewise, classical statistical inference methods have advanced in hypothesis testing and Bayesian statistics is rapidly making its way both in experimental studies and in psychometric studies. The aim of this issue has been to contribute to the dissemination of new research methodologies in quantitative and qualitative analysis in Psychology, as well as to evaluate the effectiveness and advantages of the new methods compared to classic psychometric tools and methods.

Manuscripts on methodological issues of current interest, meta-analyses or systematic reviews, articles that introduce new applications on various procedures used in solving problems in Psychology, and reviews of statistical software have been welcomed. Likewise, researchers from other areas were invited to make presentations in which the relevance for psychology of the procedures developed in other fields could be verified.

In this issue, 17 manuscripts have been submitted, of which 12 have been accepted. A total of 34 authors have participated in this special issue. We can see that four types of manuscripts have been published: (1) Development of scales and psychometric validation studies, (2) Theoretical and/or opinion articles, (3) Simulation studies, and (4) Systematic reviews or meta-analysis.

## Development of scales and psychometric validation studies

Hosseini et al. carried out a cross-sectional study with a sequential exploratory mixed method approach to develop and study the psychometric properties of a family caregivers' resilience scale in a sample of 435 family caregivers in Iran. Both the face and content validity of the new scale were studied, and an item analysis was performed using the corrected item-total correlation before studying the construct validity using both exploratory and confirmatory factor analysis. The scale also showed good reliability (composite reliability index and average variance extracted) and stability.

Nurumov et al. carried out a psychometric study of a short version of the Marlowe-Crowne Social Desirability Scale (MCSDS) in a nationally representative sample of teachers in Kazakhstan. Nurumov et al. carried out a psychometric study of a social desirability scale using a representative sample of Kazakh teachers. The Random Intercept Item Factor Analysis technique is used to study the construct validity and, although previous studies indicate that the scale is one-dimensional, the results suggest that it evaluates two correlated factors: attribution and denial. Furthermore, measurement invariance was confirmed based on geographic location, language spoken, and different age groups, but not based on gender.

## Theoretical and/or opinion articles

Stone, in an opinion article, raises several issues related to the use of fit indices in structural equation modeling. It is known, from simulation studies, that each fit index is more favorable to consider that a model fits the data under certain conditions. Therefore, researchers usually offer in their results only those fit indices that indicate a good fit of the model. Similarly, only certain fit indices are offered when the model does not fit. In addition, although certain fit indices are almost always published when performing a confirmatory factor analysis, other indices offered by authors vary greatly depending on whether or not they support their hypotheses regarding model fit. For these reasons, Stone proposes that journals should set standards for reported fit indices when submitting articles related to structural equation modeling. He also suggests that researchers use the three-step process proposed by Kline (2016) to assess the fit of a model and take into account the limitations of the fit indices themselves.

Tofighi, in his theoretical manuscript extends the correlated augmented mediation sensitivity analysis (CAMSA), to a non-randomized latent growth curve mediation model (LGCMM). Furthermore, highlights the need to establish techniques to assess the violation of the assumption of non-omission of confusion in this type of model. After demonstrating how failure to meet this assumption affects the fit of the model, he offers an R code to use in the structural equation modeling framework to perform this sensitivity analysis.

Metsämuuronen studies the underestimation of reliability caused by the inadequate calculation of the correlations between the items since, depending on the measurement scale, the estimation must be carried out in different ways. Thus, this author proposes different groups of corrected reliability estimators and studies their behavior using polychoric correlation coefficients. In this way, corrected reliability coefficients are offered, such as the Goodman-Kruskal gamma and the Somers delta.

## Simulation studies

Huang et al. carry out a simulation study framed in the Item Response Theory (IRT) using the 2-Parameter Logistic Model and the Graded Response Model, and their corresponding versions for the Testlet Response Theory (TRT) framework. Specifically, they study the violation of the assumption of local independence when groups of items (testlets) are used to construct a test, as well as its influence on the equating of scores. These simulations are carried out with both dichotomous and polytomous models, taken into account the effect of the testlet, the number of items in the testlet, and the sample size in the estimation of the item and ability parameters. The results show, under all conditions, that TRT models perform more accurately than IRT models, supporting the use of TRT models to equate tests composed of testlets. On the other hand, it was found that the IRT and TRT models worked similarly with large sample sizes.

Zhang et al. also carry out a simulation study within the IRT framework and in response time models, from a Bayesian perspective. The authors propose a new Bayesian sampling algorithm to analyze data on outlier response and response time. Simulation studies show that this algorithm accurately estimates the parameters of the studied models, although under certain conditions the estimation takes time to be obtained, so it would be desirable to develop specific software for large sample sizes.

Metsämuuronen, after performing a simulation study, proposes an initial typology of families of deflation-corrected reliability estimators. In this proposal, he has distinguished between the most suitable estimators for binary or polytomous elements, as well as whether the proposed estimators are more suitable with small or large samples.

Keck et al., perform several simulation studies to clarify type I and type II error rates of the Wald, LRT, Score, Fbar, and D statistics under different conditions. The authors recommend using the Wald and Fbar test instead of the Likelihood-Ratio Test and the Score test, since the first two require less time to be calculated. Furthermore, the authors advise using the corrected degrees of freedom instead of the mean squared error when calculating the test statistics, and using the Fbar distribution instead of the squared Chibar distribution when calculating *p*-values.

Kudrna and Kushlev have studied the relationship between income and daily happiness, since some studies, by treating income as a continuous variable, consider that there is no relationship between both variables. These authors reanalyzed existing data from the United States and Germany considering income as a categorical variable (instead of considering it continuous) and exploring non-linear relationships. The results have shown that some people with lower incomes were happier than some people with higher incomes. That is, depending on the approach, discrepant results can be obtained on the correlates of happiness. Therefore, when studying the relationship between income and subjective wellbeing, it is convenient to explain the method of analysis used to measure and analyze income.

## Systematic reviews or meta-analysis

Yoon et al. carry out a meta-analysis to study the psychometric properties of Gaming Disorder Scales (GD). Five representative GD instruments were included, with 2,124 studies full text assessed, and 184 quantitatively synthesized. Results show that all five GD instruments showed good internal consistency and test-retest reliability. The result of the meta-analysis showed that all the scales analyzed had good reliability. Likewise, gaming disorder scores showed moderate correlations with game time and with other associated problems (anxiety and depression). However, there was a strong correlation with Internet addiction.

Nia et al. carried out a systematic review of the psychometric properties of hardiness scales using the COSMIN checklist and the Terwee quality criteria. In total 33 articles were entered in their study. It was found that construct validity was offered in almost all of them, and content validity and internal consistency were also reported. In addition, 12 studies provided information on cross-cultural invariance. Finally, four were the best measurement instruments to assess hardiness in different types of population.

This Research Topic includes 12 very different articles, since there are statistical simulations, systematic reviews, psychometric studies, as well as opinion and theoretical articles. We hope that all of them will contribute to new research methodologies in the Social Sciences.

## Author contributions

All authors listed have made a substantial, direct, and intellectual contribution to the work and approved it for publication.
